# The association between patient age, abscess size, and white blood cell count on duration of catheter stay for percutaneous abscess drainage of abdominal abscesses

**DOI:** 10.1186/s13104-024-06954-x

**Published:** 2024-10-07

**Authors:** Christopher Stevens, Chintan Mehta, Dylan Scott, Prerana Ramesh, Amanda Ragland, Coplen Johnson, Joshua Strobel, Christopher Schmoutz, Assala Aslan, Chaitanya Ahuja, Luis De Alba

**Affiliations:** 1 Department of Radiology, Louisiana State University Health Sciences Center-Shreveport, 1501 Kings Highway, Shreveport, Louisiana, LA 71103 USA; 2 Department of Toxicology, Louisiana State University Health Sciences Center-Shreveport, 1501 Kings Highway, Shreveport, Louisiana 71103 USA

**Keywords:** Abscess, Percutaneous, Catheter, Abdomen, Interventional Radiology

## Abstract

**Objective:**

Knowing factors that impact catheter stay duration is important since removing drainage catheters too early or late can have significant consequences. We present a single center retrospective study that analyzes multiple variables, including abscess size, white blood cell count, and patient age, to see if a correlation between them and duration of catheter stay exists. The inclusion criteria were patients that had abdominal abscesses treated with percutaneous abscess drainage using a pigtail catheter, ≥ 18 years of age, and had available medical images and records. 44 patients were included.

**Results:**

Among white blood cell count, patient age, and abscess volume, the only significant relationship with duration of catheter stay was abscess size (*R* = 0.42, p-value = 0.0049).

## Introduction

An abscess is described as a collection of purulent material that usually forms from infection induced inflammation. Most abscesses, no matter the location, require drainage [1]. Percutaneous abscess drainage (PAD) is a minimally invasive procedure performed commonly by interventional radiologists and involves using image guidance to deploy a drainage catheter into the abscess by perforating the skin and overlying soft tissues [1]. Despite the increase in PAD usage, there are still some unanswered questions and absent guidelines regarding its use, including how long a catheter should stay in place for and certain variables that might affect this.

Herein, we present a single center retrospective study that analyzes multiple variables, including size of abscess, white blood cell (WBC) count, and patient age, to see if a correlation between them and duration of catheter stay existed. Our hypothesis was that there would be a significant positive correlation in all three relationships. Regarding abscess size, we suspected a positive relationship due to a larger abscess having more volume and thus taking longer to drain. For WBC count, we hypothesized that a higher WBC count would lead to greater duration of catheter stay due to WBC count having a direct relationship with the degree of infection and the body taking longer to clear more vigorous infections. We believed a significant positive correlation would exist between age and catheter duration since it takes older individuals longer to heal and clear infections from the body compared to younger people and them also having a higher chance of experiencing impaired recovery following infection.

## Methods and materials

### Study design and patient selection

This study was approved by the Institutional Review Board (STUDY00002183). For this single center retrospective study, Epic Slicer-Dicer was used to recruit patients from January 1, 2020, to December 31, 2023, at our institution. The inclusion criteria for this study were patients that had abscesses treated with PAD using a pigtail catheter, *≥* 18 years of age, and had available medical images and medical records within in the timeframe described previously. Location of abscesses was limited to the abdomen only. The exclusion criteria consisted of patients who had multiple drainage catheters placed simultaneously or insufficient follow up notes. 44 patients met the criteria and were included in the study.

### Procedures

We obtained age, sex, location and size of abscess, size of drainage catheter, length of drainage catheter stay, and WBC count before catheter placement. Abscess volume was quantified by using the ellipsoid formula: 4/3 π · (rx) · (ry) · (rz), where *V* is the volume, and *r* represents the radius of the abscess in three different planes, *x*, *y*, and *z.* This formula operates under the principle that abscesses are spherical or ellipsoid [2]. Measurements for *x*, *y*, and *z* were collected by measuring the size of the abscess on computed tomography (CT) and the volume of the abscess was expressed as cm^3^. WBC count was expressed as K/uL.

### Analysis

Patient data were analyzed for assumptions of equal variance, normality, and linearity. One data point was identified as an outlier and excluded from the final analyses, thus leaving a total of 43 patients included in the results. Correlation coefficients between identified variables were obtained by using Pearson correlation analysis, and a p-value ≤ 0.05 was considered significant. All analyses and visualizations were performed using R Statistical Software (v4.3.3; R Core Team 2024).

## Results

**Fig. 1:**
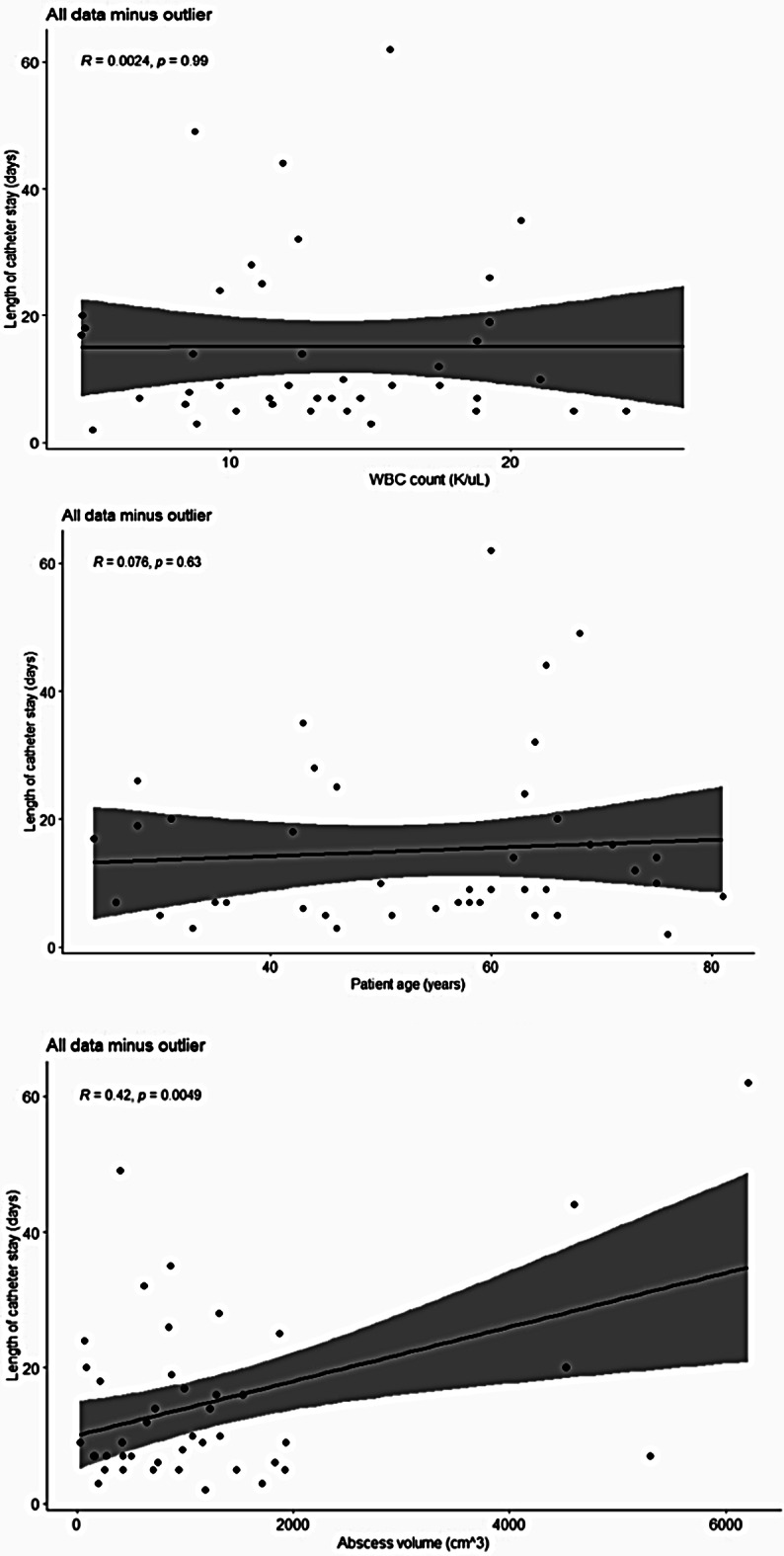
Scatter plot depicting the correlation between duration of catheter stay for all catheter sizes and (**a**) WBC count, (**b**) patient age, and (**c**) abscess size

Figure 1 express the relationships between WBC count, patient age, and abscess volume with duration of catheter stay for all 43 patients.

**Fig. 2:**
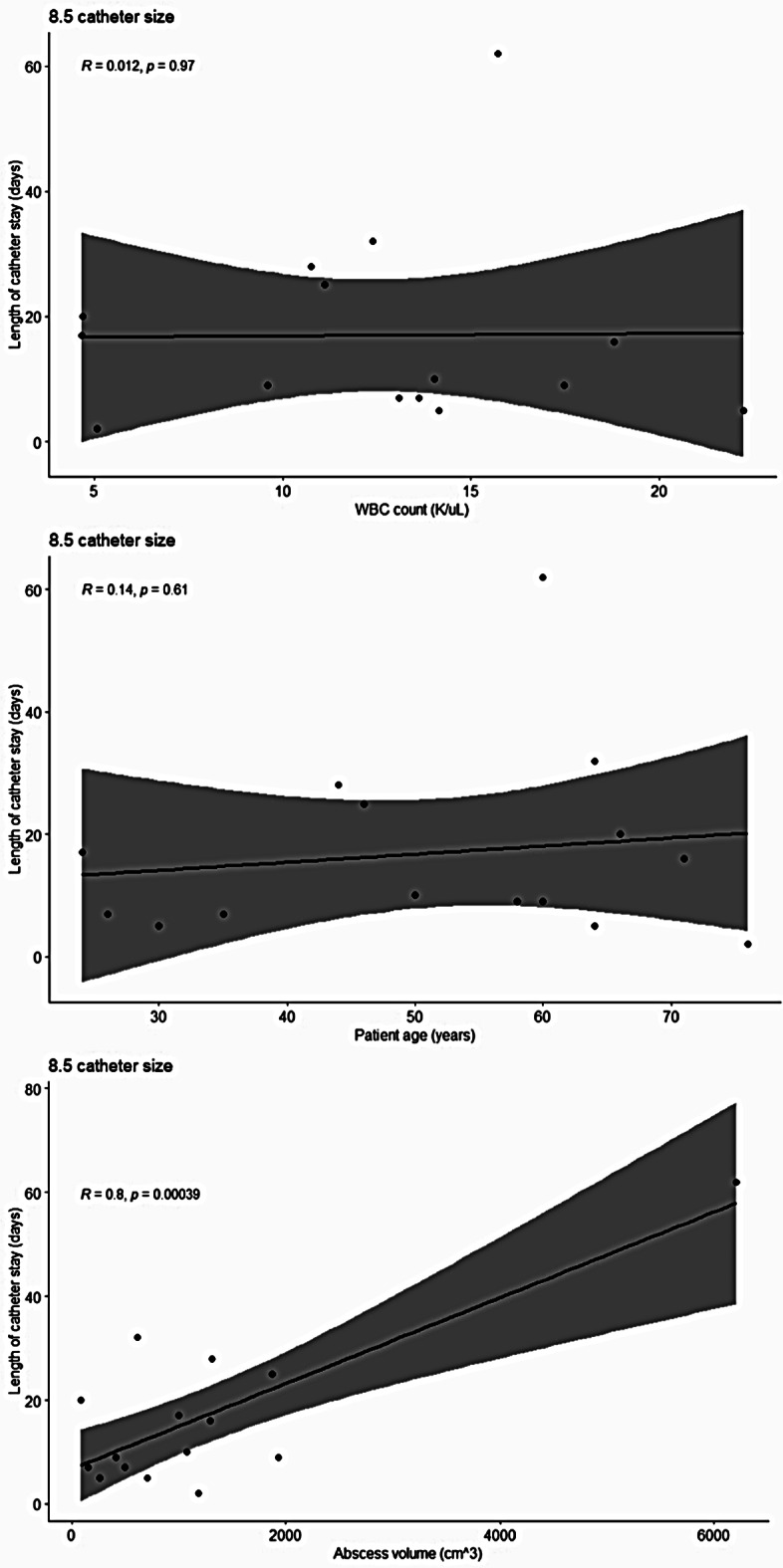
Scatter plot depicting the correlation between duration of catheter stay for 8.5 F sized catheters and (**a**) WBC count, (**b**) patient age, and (**c**) abscess size

Among WBC count, patient age, and abscess volume, the only significant relationship with duration of catheter stay was abscess size (*R* = 0.42, p-value = 0.0049). Not shown are catheter sizes used, which included 8 French (F), 8.5 F, 10 F, and 12 F, with 8.5 F being the most common (*n* = 15). Since 8.5 F was the most common, we decided to analyze the same three relationships as shown in the figure above but with only the 8.5 F catheter (Fig. 2). This allowed for retirement of catheter size as a potential variable that might have affected the duration of catheter stay in the relationships shown in Fig. 1.

Like the data in Fig. 1, the only significant relationship associated with use of 8.5 F catheters was that of abscess volume (*R* = 0.8, p-value = 0.00039).

## Discussion

This brief retrospective study adds to the literature by showing that abscess volume, when compared to WBC count and age of patient, is the leading factor that influences how long a catheter stays deployed for. Knowing factors that impact duration of catheter stay is important since removing drainage catheters too early or late can have significant consequences. Removing the catheter before the abscess has been completely drained can lead to recurrence of the fluid collection, but leaving the catheter in for extended durations may cause skin infection at the catheter site [3].

An exact guideline for how long a drainage catheter should stay in place for is still lacking in the literature; however, it has been said drainage catheters can be removed when: the drainage output is reduced to 10–20 mL/day or less, the size of the abscess cavity is reduced, abscess-related fistulas are absent or resolved, and the patient’s vital signs return to normal levels [4]. Regular monitoring of the patient, the fluid drainage output, and obtaining the necessary follow-up tests can help minimize complications and identify modifications to be made, such as adjustment of the patients’ medications and replacement of the catheter if necessary.

## Limitations

While this manuscript discusses novel findings in the realm of an important topic, limitations do exist. One was that it is a single center study with a limited number of subjects, thus leading to a decrease in variability of the population studied. Another limitation is that not all the catheters were placed and removed by the same radiologists, meaning personal preference for when the radiologist decided to remove the catheter could have potentially led to variable results; however, this also highlights the notion that there is no exact guideline for physicians to follow regarding when a drainage catheter should be removed. This is a topic that future studies can explore since removing a drain to early or too late can potentially have significant consequences. Second, not all abscess sizes were measured by the same radiologist, meaning there was a lack of standardization in this aspect of the study. Lastly, the authors did not include the timing of the PAD insertion as a variable in this study. The natural course of the patient’s diseased state, when combined with the high probability that not all patients had the PAD insertion occur at the same timeframe of their condition, could have affected the treatment duration and PAD stay. The authors did not include when PAD was initiated in the timeframe of each patients’ condition because many patients had inadequate notes in their charts that made it difficult for the authors to find an accurate time as to when each patient’s condition began.

## Conclusion

In this study, we retrospectively analyzed certain factors, specifically patient age, WBC count, and abscess volume, to see if there was a correlation between them and duration of catheter stay. We found that patient age and WBC showed no significant correlation while abscess volume showed a strong positive correlation associated with length of catheter stay.

## Data Availability

The datasets used and/or analyzed during the current study are available from the corresponding author on reasonable request.

## Abbreviations

PAD: Percutaneous abscess drainage
WBC: White blood cell
CT: Computed tomography
F: FRENCH
